# Experiences of moderate-to-severe demoralization syndrome among patients receiving maintenance hemodialysis: a qualitative study

**DOI:** 10.3389/fpsyg.2026.1931026

**Published:** 2026-10-15

**Authors:** Jianfang Bao, Yijia Yin, Xuan Cheng, Xiaoxue Jiang, Binhong Luo, Suping Guo, Jun Xu, Min Qian

**Affiliations:** 1 School of Nursing, Medical Department, Yangzhou University, Yangzhou, Jiangsu, China; 2 Northern Jiangsu People's Hospital, Yangzhou, Jiangsu, China

**Keywords:** demoralization syndrome, maintenance hemodialysis, patient experience, psychological distress, qualitative research

## Abstract

**Background:**

Patients receiving maintenance hemodialysis (MHD) are at high risk of demoralization syndrome (DS), which may adversely affect psychological wellbeing, quality of life, and treatment-related self-management. However, limited qualitative evidence exists regarding their experiences.

**Aim:**

To explore the experiences of moderate-to-severe DS among patients receiving MHD, focusing on stressors, psychological and behavioral experiences, coping strategies, and stress-buffering resources.

**Methods:**

A descriptive qualitative design guided by the Stress Process Model (SPM) was adopted. Purposive maximum-variation sampling was used to recruit 15 patients receiving MHD with moderate-to-severe DS (DS-II score >9) from a tertiary hospital in China between January and March 2026. Data were collected through semistructured, face-to-face, in-depth interviews. All interviews were audio-recorded and transcribed verbatim, and the transcripts were verified by participants. Data were analyzed using thematic analysis following Braun and Clarke’s six-phase framework.

**Results:**

Four framework-informed themes and 14 subthemes were identified, corresponding to the major SPM domains of sources of DS, impacts of DS, coping strategies, and stress-buffering resources. Two important distinctions were identified: reduced social participation primarily reflected illness- and treatment-related restrictions, whereas social withdrawal represented a more intentional behavioral response associated with greater demoralization; and apparent acceptance of dialysis sometimes reflected helpless resignation and passive compromise rather than adaptive acceptance.

**Conclusion:**

DS among patients receiving MHD reflects a complex process shaped by interacting treatment-related stressors, coping responses, and social support resources. Interventions should aim to reduce modifiable stressors, strengthen adaptive coping and perceived control, and support psychological adaptation, functioning, and meaningful engagement during long-term dialysis.

## Introduction

Chronic kidney disease (CKD) represents a major global health challenge and may progress to end-stage kidney disease (ESKD), requiring long-term renal replacement therapy (Ene-Iordache et al., 2016; Thurlow et al., 2021). Among available treatment modalities, maintenance hemodialysis (MHD) remains the most commonly used approach for sustaining life in patients with ESKD (Torreggiani et al., 2023). Although advances in dialysis technology have improved survival outcomes, MHD patients continue to experience substantial treatment-related burdens, including persistent physical symptoms, dietary and fluid restrictions, dependence on regular dialysis schedules, and disruptions in social and occupational functioning (Sluiter et al., 2024). These long-term challenges require continuous psychological adaptation and may place patients at increased risk of psychological distress and maladaptive adjustment (Cao et al., 2025).

Patients receiving MHD frequently experience psychological difficulties, including depression, anxiety, uncertainty about the future, and reduced quality of life (Zhang et al., 2024). However, these psychological responses do not fully capture the existential challenges associated with living with a life-sustaining treatment. In particular, demoralization syndrome (DS) has received increasing attention as a clinically significant psychological condition among patients with chronic and life-threatening illnesses (Zhang et al., 2025). DS was initially conceptualized by Clarke and Kissane (2002) as a state of existential distress characterized by helplessness, hopelessness, loss of meaning, and a perceived inability to cope with stressful life circumstances (Clarke and Kissane, 2002; Lv et al., 2025). Unlike depression, which is primarily characterized by persistent depressed mood, anhedonia, and diminished interest or pleasure, DS is more specifically associated with loss of purpose, reduced perceived competence, and feelings of failure in coping with ongoing challenges (Kaneriya et al., 2023). Individuals experiencing DS may still retain emotional responsiveness and the ability to experience pleasure, but they struggle with a diminished sense of meaning, control, and future orientation (Hua and Bhatarasakoon, 2025). Demoralization and depression are conceptually related but distinguishable forms of psychological distress. Previous research in Chinese patients has shown that the two constructs are associated, while some patients may exhibit high demoralization in the context of relatively low depressive symptoms (Wu et al., 2021b). However, substantial overlap may occur in experiences such as hopelessness, negative affect, reduced interest, and social withdrawal. Because depression was not independently assessed in the present study, we cannot determine whether these overlapping experiences were attributable specifically to demoralization or to co-occurring depressive symptoms. Accordingly, this study was not designed to assess the discriminant validity of demoralization relative to depression. Rather, participants were selected on the basis of DS-II scores indicating moderate-to-severe demoralization, and the qualitative analysis focused on how they described the meanings, perceived coping difficulties, loss of agency, valued-role disruption, and future-oriented concerns associated with their experiences. These dimensions were therefore interpreted as salient features of participants’ accounts of demoralization rather than as evidence that depressive symptoms were absent.

Although DS overlaps phenomenologically with other forms of psychological distress, its core features are characterized by excessive worry, fear, and anticipation of future threats, whereas hopelessness refers mainly to negative expectations regarding future outcomes (Marchetti et al., 2023; Ohi et al., 2025). Existential distress broadly involves concerns related to meaning, identity, mortality, and one’s place in life (Vehling and Kissane, 2018). In contrast, demoralization places particular emphasis on the interaction between perceived inability to cope with current stressors and the erosion of meaning, agency, and future orientation (Clarke and Kissane, 2002). Accordingly, the present study treats these features as interpretive dimensions of participants’ experiences rather than as evidence of discriminant validity from depression.

DS is commonly reported among patients with cancer, advanced chronic diseases, and other medically vulnerable populations (Chang et al., 2022; Shu et al., 2025). Previous quantitative studies have demonstrated that moderate-to-severe DS is prevalent among MHD patients and is associated with psychological burden, impaired quality of life, and difficulties in treatment adaptation (Kissane, 2001; Zhang et al., 2025). However, existing research on DS in MHD populations has predominantly relied on quantitative approaches, which provide limited understanding of how patients experience demoralization, interpret their illness-related challenges, and respond to the psychological consequences of long-term dialysis (Lv et al., 2025; Zhang et al., 2025). A deeper exploration of patients’ experiences is therefore needed to understand the contextual factors associated with DS and to inform the development of targeted interventions.

The Stress Process Model (SPM), proposed by Pearlin et al. (1990) provides a useful theoretical framework for understanding psychological adaptation under chronic stress. The model conceptualizes stress as involving stressors, mediating processes such as coping strategies and social support, and psychological outcomes. In the context of MHD, persistent treatment burden, uncertainty, lifestyle disruption, and changes in social roles may represent important stressors, while coping strategies and available support resources may influence patients’ psychological experiences. However, few studies have applied the SPM to explore DS among patients receiving MHD from a qualitative perspective.

Beyond its emphasis on stress exposure and psychological distress, the SPM can also be connected conceptually with perspectives on resilience and perceived control. Resilience is commonly understood as a dynamic process of adapting to adversity and maintaining or restoring functioning despite ongoing stressors (Chmitorz et al., 2018; Morse et al., 2021). Within this perspective, the SPM constructs of coping and stress-buffering resources may represent protective processes that help individuals maintain functioning and adapt to chronic illness. Perceived control provides an additional link between these perspectives, as maintaining a sense that one can influence or manage important aspects of life may facilitate active coping and psychological adaptation under chronic stress (Ranchor et al., 2010; Meier et al., 2026). In the context of MHD, these perspectives complement the SPM by highlighting not only how stress accumulates, but also how patients may preserve agency, meaning, and future orientation while adapting to long-term treatment.

Therefore, guided by the SPM, this study employed a descriptive qualitative design to explore the experiences of moderate-to-severe DS among MHD patients. Specifically, this study aimed to examine patients’ perceived stressors, psychological and behavioral experiences, coping strategies, and stress-buffering resources related to DS, thereby providing insights for the development of comprehensive psychological care strategies for this population.

## Methods

### Study design

This study adopted a descriptive qualitative design and was reported in accordance with the Consolidated Criteria for Reporting Qualitative Research (COREQ) guidelines to ensure methodological rigor and transparency (Tong et al., 2007).

A descriptive qualitative design was selected because the aim of this study was to obtain a comprehensive and straightforward description of patients’ experiences related to moderate-to-severe demoralization within the context of MHD, rather than to interpret the essence of lived experience or develop a philosophical understanding of the phenomenon. This approach is particularly appropriate for exploring clinically relevant experiences and identifying common patterns from participants’ accounts while remaining close to their descriptions.

### Setting and participants

This study was conducted between January and March 2026 in the Hemodialysis Center of a tertiary-level public hospital in Yangzhou, Jiangsu Province, China, which serves as a regional referral center for nephrology care and provides comprehensive dialysis services to both urban and rural populations. The center maintains a stable cohort of long-term MHD patients.

A purposive maximum-variation sampling strategy was adopted to ensure heterogeneity in age, gender, educational level, dialysis duration, and primary renal disease. Participants were initially identified from a previous cross-sectional survey conducted by the research team in the same clinical setting. In the parent survey (Luo et al., 2026), demoralization, personal mastery, self-efficacy, and coping styles were assessed using the Demoralization Scale-II (DS-II) (Robinson et al., 2016), Personal Mastery Scale, General Self-Efficacy Scale, and Medical Coping Modes Questionnaire, respectively. No depression-specific instrument was included in the parent survey. Therefore, depressive symptoms or clinically significant depression were not specifically assessed. The DS-II is a 16-item self-report instrument, with each item rated on a 3-point scale from 0 to 2, yielding a total score ranging from 0 to 32, with higher scores indicating greater demoralization severity (Robinson et al., 2016). In the original validation study, the DS-II was established as a continuous measure of demoralization severity; subsequent studies, including research in Chinese patients receiving maintenance hemodialysis, have used score bands of ≤9, 10–19, and ≥20 to indicate mild, moderate, and severe demoralization, respectively. In the present study, a DS-II score >9 was used as the eligibility criterion to identify patients with at least moderate levels of demoralization, thereby including both moderate (10–19) and severe (≥20) presentations (Wu et al., 2021a). This cutoff has also been used in previous studies of Chinese patients and has demonstrated applicability in maintenance hemodialysis populations (Zhou et al., 2023).

Among the 245 MHD patients screened in the previous cross-sectional survey, 83 patients scored >9 on the DS-II and were considered potential candidates for the qualitative study. After further assessment according to the eligibility criteria, all 83 patients met the initial eligibility criteria. To achieve maximum variation in age, sex, educational level, dialysis duration, and primary renal disease, 36 of the 83 eligible patients were purposively selected and invited in person by clinical staff to participate in the qualitative interviews. Of these, 16 patients agreed to participate, whereas 20 declined. The reasons for non-participation included lack of time (*n* = 5), physical discomfort (*n* = 8), and unwillingness to discuss personal experiences (*n* = 7). Although maximum-variation sampling was used to seek heterogeneity in age, sex, educational level, dialysis duration, and primary renal disease, the final variation achieved was also shaped by patients’ willingness and ability to participate.

Because approximately 5 months had elapsed between the previous cross-sectional survey and the qualitative interviews, eligibility was reassessed before interviewing using the DS-II. Of the 16 patients who agreed to participate, one no longer met the DS-II eligibility threshold (score ≤9) and was excluded. The remaining 15 patients met the eligibility criteria and were included in the final sample. Therefore, the DS-II scores presented in Table 1 were derived from the pre-interview re-screening conducted before qualitative interviews. The participant recruitment and selection process is presented in Figure 1.

**Table 1:** Sociodemographic and clinical characteristics of the study participants (*n* = 15).

Participant	Gender	Age (years)	Work status	Marital status	Education	Duration of dialysis (years)	Primary disease	DS-II score^1^
P1	Female	41	Employee	Married	College degree or above	8	Hypertension	16
P2	Male	42	Unable to work due to illness^2^	Divorced	College degree or above	2	Polycystic Kidney	12
P3	Female	43	Unemployed	Married	Junior high school	7	Hypertension	25
P4	Female	49	Unemployed	Married	Senior high school	8	Diabetes	10
P5	Female	61	Retired	Married	Junior high school	1	Hypertension	17
P6	Female	63	Retired	Married	Junior high school	6	Diabetes	15
P7	Male	40	Employee	Married	College degree or above	2	Polycystic kidney	27
P8	Male	54	Self-employed	Married	Junior high school	1	Hypertension	11
P9	Male	50	Unable to work due to illness^2^	Divorced	Senior high school	1	Polycystic kidney	14
P10	Female	64	Retired	Married	College degree or above	10	Diabetes	10
P11	Male	39	Unable to work due to illness^2^	Never married	College degree or above	5	Glomerulonephritis	20
P12	Male	71	Retired	Married	Junior high school	13	Glomerulonephritis	23
P13	Male	53	Unemployed	Married	Junior high school	3	Polycystic kidney	18
P14	Male	49	Unable to work due to illness^2^	Married	Junior high school	9	Glomerulonephritis	12
P15	Female	44	Unable to work due to illness^2^	Divorced	College degree or above	4	Hypertension	24

^1^DS-II = Demoralization Scale-II. ^2^Unable to work due to illness indicates that participants discontinued employment because of health-related limitations associated with chronic kidney disease and dialysis treatment rather than age-related retirement.

**Figure 1:**
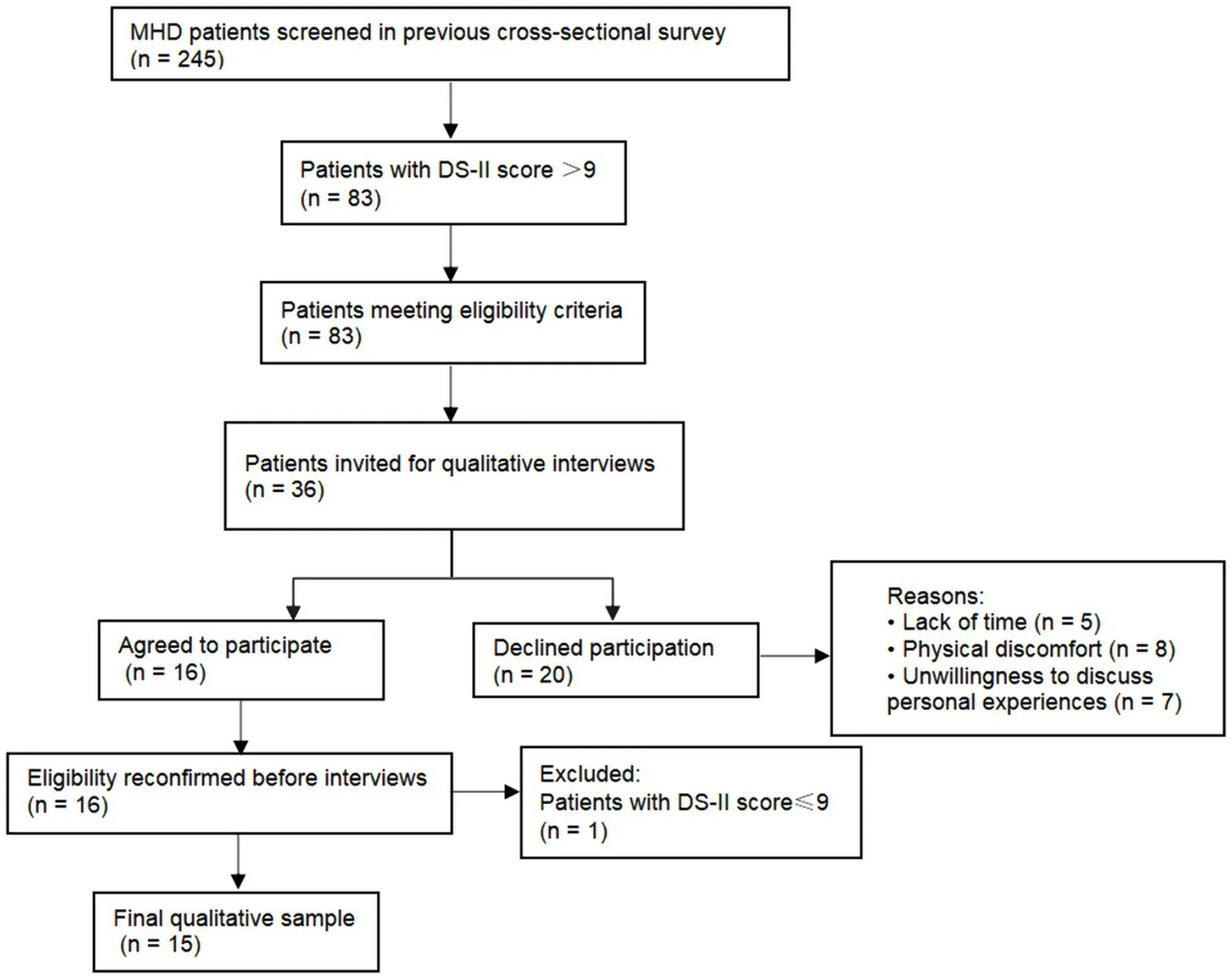
Participant recruitment pathway.

Data saturation was considered achieved after 13 interviews when no new themes emerged. Two additional interviews were subsequently conducted to confirm thematic redundancy. Therefore, a total of 15 participants were included in the final analysis. The general demographic characteristics of the participants are shown in Table 1. The inclusion criteria were as follows: (1) age ≥18 years; (2) dialysis duration ≥3 months (Chen et al., 2021); (3) DS-II score >9; (4) ability to communicate verbally; and (5) willingness to participate and provide written informed consent (Chen et al., 2021). Exclusion criteria were: (1) acute medical instability (e.g., acute infection or preparation for kidney transplantation); and (2) clinically diagnosed severe psychiatric disorders or cognitive impairment.

### Instrument

The semistructured interview guide was developed based on the SPM and integrated three core domains: stressors, mediating processes (coping strategies and social support), and stress outcomes (Pearlin et al., 1990). The initial draft was informed by a literature review and discussions among the research team. To enhance its applicability and comprehensibility, a preliminary pilot test was conducted with two MHD patients. Based on their feedback, minor modifications were made to improve the wording and sequence of several questions. These two patients participated only in refinement of the interview guide and were not included in the final study sample. The revised guide was subsequently reviewed by a panel of three experts in nursing, psychology, and medical ethics. The final version was established after their suggestions were incorporated (Table 2). The complete interview guide is provided in Supplementary File S1.

**Table 2:** Semistructured interview guide.

1. Could you share your experiences since starting dialysis? How has dialysis changed your life?
2. During your dialysis treatments, what have been the most helpless, frustrating, or demoralizing moments? Could you describe these feelings in more detail?
3. How have these feelings impacted your life? How do you cope with and manage these psychological reactions?
4. What do you believe these feelings are related to?
5. At this stage, what challenges are you facing? What is the biggest challenge for you?
6. How do you think the people around you (family, friends, or healthcare professionals) can help? Are there any other thoughts or feelings you’d like to share?
7. What advice would you offer to those who are just starting dialysis? What would you like to say to them?
8. Is there anything else you would like to add or share that we have not covered?

## Data collection

Data were collected through face-to-face, semistructured, in-depth interviews conducted by two trained postgraduate nursing students (B. J. F. and J. X. X.). Both researchers had received formal training in qualitative research methods and were familiar with qualitative interviewing principles, procedures, and techniques. They also participated in the development of the interview guide and were familiar with the study objectives and theoretical framework before data collection. The first researcher (B. J. F.) conducted the majority of the interviews, while the second researcher (J. X. X.) conducted the remaining interviews.

No prior contact or therapeutic relationship existed between the researchers and participants. Before each interview, the researchers established rapport with the participants, explained the study purpose, procedures, and ethical considerations, and obtained written informed consent from the participants. Participant safety procedures were specified in the ethics-approved study protocol (Approval: 2025ky369). During the interviews, researchers monitored participants for marked emotional distress and expressions of self-harm or suicidal ideation. When clinically concerning distress or suicidal ideation was identified, the interview was stopped, and the participant was immediately referred to the responsible nurses and physicians for further clinical assessment and management. The safety procedure was activated for two participants. For P10, it was activated when the participant expressed thoughts indicating that they should not be alive during the discussion of persistent negative emotions. For P13, it was activated when the participant stated during the discussion of dialysis-related physical discomfort that the pain was so severe that they sometimes did not want to live anymore. In both cases, the interview was stopped immediately and was not resumed. After the safety procedure had been activated, the researchers reconfirmed with each participant that the interview material collected before activation could be retained and used for the present study; both participants agreed. Accordingly, only the material collected before activation was retained and analyzed, and no additional interview data were collected after the safety procedure was initiated. The responsible clinical team was informed and assumed responsibility for subsequent assessment and management according to routine hospital procedures. The research team did not collect or analyze subsequent clinical management or follow-up outcomes. Interviews were conducted in a quiet, private, and undisturbed environment in a teaching room within the hospital. Only the participant and interviewer were present during each interview, and no family members, caregivers, or other healthcare professionals were present. All interviews were conducted after completion of participants’ dialysis sessions on scheduled dialysis days. This timing was selected to avoid interfering with dialysis care and to allow participants to reflect on their recent treatment experiences after completing their treatment procedures.

No repeat interviews were conducted. The entire conversation was audio-recorded with the participants’ consent. While the interview guide provided a framework, flexibility was maintained to adapt the sequence and format of questions to delve deeper into participants’ narratives. In addition to recording verbal responses, the researchers made real-time notes during each interview to document non-verbal behaviors and emotional expressions, such as crying, laughter, anger, or anxiety. The duration of interviews varied from 30 to 60 min, depending on participants’ willingness and physical stamina to engage in extended conversation. After each interview, the data (audio files and notes) were assigned a unique participant code (e.g., P1–P15), and reflective diaries and observational notes were written.

Data saturation was assessed iteratively during data collection and analysis. After each interview, the research team conducted preliminary coding and discussed emerging codes, categories, and themes. Saturation was considered to have been reached after the 13th interview, when no substantively new codes, themes, or insights relevant to the research questions emerged. Two additional interviews were subsequently conducted to confirm thematic redundancy and the stability of the identified themes, resulting in a final sample of 15 participants.

### Data analysis

Within 24 h after each interview, two researchers independently transcribed the audio recordings verbatim. The transcripts were then returned to participants for verification and correction when necessary. Data collection and analysis were conducted concurrently. Qualitative data were analyzed using NVivo 12.0 software, and thematic analysis was performed following the six-phase framework proposed by Braun and Clarke (2006). Given that the SPM informed the interview guide and provided the conceptual structure for the study, a deductive, framework-informed thematic analysis was adopted. The major SPM domains were used as the primary framework for organizing and interpreting the data, including stressors, psychological and behavioral impacts, coping strategies, and stress-buffering resources. The analysis followed Braun and Clarke’s six-phase framework: (1) familiarization with the data through repeated reading of transcripts; (2) generation of initial codes related to the research questions and relevant SPM domains; (3) organization of related codes into preliminary categories; (4) review of candidate themes against the dataset; (5) definition and naming of themes and subthemes; and (6) production of the final report using representative quotations. Two researchers independently coded and analyzed the transcripts, and any disagreements were resolved through discussion until consensus was reached or through consultation with a senior qualitative research expert. This iterative process enhanced the credibility and rigor of the qualitative analysis.

### Trustworthiness

Multiple strategies were employed to ensure the trustworthiness of the study, including investigator triangulation, member checking, peer debriefing, and an audit trail. Two researchers independently coded and analyzed the data, and discrepancies were resolved through iterative discussion to reach consensus on the final themes. Transcript verification and member checking of preliminary themes were conducted as two separate procedures. After transcription, six participants with diverse demographic and clinical characteristics reviewed their transcripts to verify accuracy and identify potential misunderstandings. Participants confirmed that the transcripts accurately reflected their experiences, and no substantive modifications were required. Subsequently, preliminary themes were shared with the same six participants to assess whether the interpretations were consistent with their experiences. Participants indicated that the themes adequately represented their experiences, and no changes were made to the final codes or themes. Peer debriefing was carried out through regular research team meetings to review the analytical process and emerging interpretations, providing an external perspective and reducing potential researcher bias. An audit trail was maintained throughout the study, including detailed documentation of interviews, coding decisions, analytical memos, and meeting records, ensuring transparency and dependability.

Reflexivity was incorporated throughout the research process to acknowledge and manage the potential influence of researchers’ backgrounds, assumptions, and clinical experiences on data collection and interpretation. The first and second interviewers were nursing postgraduate students with formal training in qualitative research methods and previous clinical exposure to hemodialysis care. Their clinical experiences provided contextual understanding of MHD patients’ physical and psychological challenges but also carried the potential risk of interpreting participants’ experiences through a clinical lens. Before data collection and during analysis, researchers engaged in reflexive discussions and maintained reflective diaries to identify personal assumptions, emotional responses, and emerging interpretations.

During interviews, researchers adopted a nonjudgmental and exploratory stance, using open-ended questions and follow-up prompts to encourage participants to describe their experiences in their own words rather than confirming researchers’ expectations. During coding and theme development, researchers repeatedly reviewed original transcripts and discussed whether interpretations were sufficiently grounded in participants’ narratives. Although the SPM provided the primary analytic framework, researchers remained attentive to variations and meaningful distinctions within each framework domain during interpretation.

### Ethical considerations

The study was conducted in accordance with the Declaration of Helsinki and was approved by the Medical Ethics Committee of the hospital (Approval: 2025ky369). All participants provided written informed consent before participation. The researchers explained the study’s purpose and procedures and assured participants of confidentiality and privacy protection. Participants were informed that they could pause or terminate the interview at any time if they experienced emotional distress.

## Results

A total of 15 participants were included in this study, of whom 46.7% were female. The mean age of the participants was 50.9 years. Regarding marital status, 11 participants were married, 3 were divorced, and 1 had never married. Educational levels ranged from junior high school to college degree or above. The duration of dialysis ranged from 1 to 13 years (Table 1).

The analysis identified four framework-informed themes and 14 subthemes corresponding to the major domains of the SPM: sources of DS, impacts of DS, coping strategies, and stress-buffering resources (Figure 2). Two distinctions were particularly important within these domains. First, reduced social participation primarily reflected illness- and treatment-related restrictions that limited opportunities for social engagement, whereas social withdrawal represented a more intentional behavioral response associated with more pronounced demoralization. Second, apparent acceptance of dialysis did not necessarily indicate adaptive acceptance; for six participants, it reflected helpless resignation and passive compromise in the context of limited perceived alternatives and prolonged illness-related stress.

**Figure 2:**
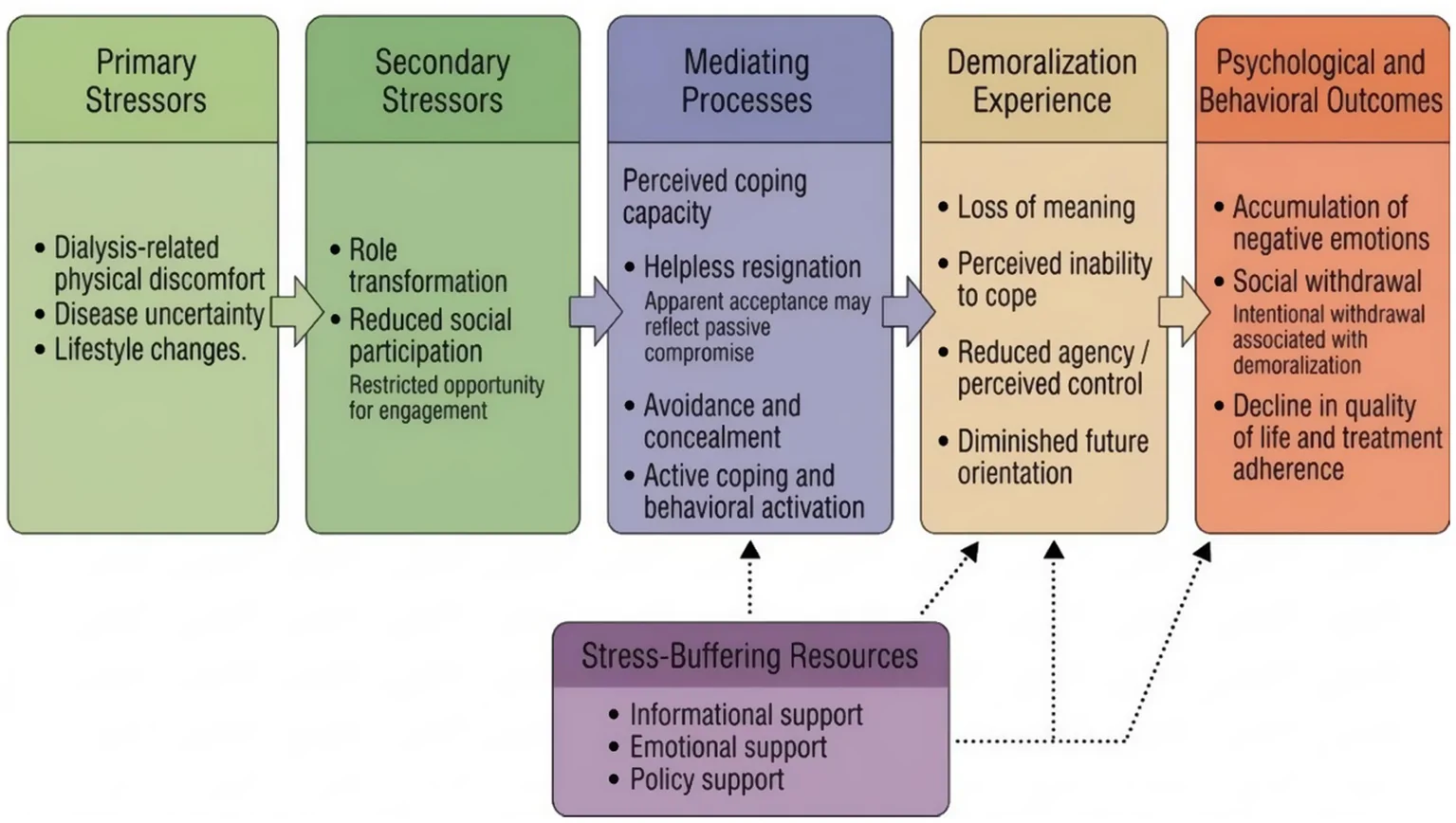
Process-oriented conceptual framework of demoralization among patients receiving maintenance hemodialysis.

## Sources of demoralization syndrome

Participants described demoralization as emerging from the accumulation and interaction of multiple stressors over time, rather than from a single triggering factor. These stressors included physical burden, disease uncertainty, lifestyle disruption, role loss, and reduced social participation.

*Dialysis-related physical discomfort*. Dialysis treatment and its associated complications represented the most direct and unavoidable physical stressors for participants. These recurring physical symptoms affected daily functioning and limited participants’ ability to engage in daily activities. Eight participants reported feeling fatigued after dialysis, making it difficult to engage in social activities beyond treatment. “I’ve been on dialysis for over a year, and my insomnia is severe. Sometimes, I only sleep for half an hour to one hour a day. I’m afraid I might collapse on the street.” (P6) Six participants mentioned experiencing discomfort such as palpitations, itching, hypotension, and muscle cramps during or after dialysis, which left them feeling physically depleted. “My skin itches and is dry; I have to keep scratching, even at night, and I feel mentally exhausted, with no motivation to do anything.” (P11) “I have a strong reaction to dialysis, with nausea, muscle cramps, and leg weakness. It’s so painful that sometimes I feel like I don’t want to live anymore.” (P13) The cumulative physical burden restricted participants’ daily activities and reduced their capacity to actively cope with ongoing illness-related challenges.

*Disease uncertainty*. Uncertainty about disease outcomes was a significant psychological stressor throughout the dialysis process. Seven participants stated that despite receiving regular dialysis, the future course of the disease remained uncertain, contributing to ongoing anxiety and helplessness. “I’ve been on dialysis for so many years, but what will happen in the future? No one can say for sure, and I feel uneasy all the time.” (P9) “A fellow patient passed away because they couldn’t manage their fluid intake. I worry if I’ll end up like them (crying).” (P4) Six participants reported that the inability to control the disease and a lack of knowledge about their condition led to a loss of control over their lives, intensifying fears about the future. “Sometimes when I feel really bad, I don’t want to take the medication or go to the hospital. It’s so hard.” (P9) “It’s confusing; dialysis feels like a bottomless pit. I just need dialysis to survive, and I have to live like this for the rest of my life.” (P14) Persistent uncertainty was accompanied by ongoing psychological distress and difficulty maintaining positive expectations for the future.

*Lifestyle changes*. Dialysis treatment led to substantial changes in daily life, disrupting participants’ established routines and contributing to feelings of frustration and restriction. First, employment was affected. The need for regular dialysis led to changes in participants’ occupational status, increasing psychological stress. “I can’t travel for work, and I can’t go to places far away.” (P1) “Dialysis has affected my workload. After I got sick, the company’s attitude changed, and the prospects for my job were gone.” (P7) Second, strict restrictions were placed on dietary and fluid intake. Participants had to restrict their intake of potassium, phosphorus, and sodium, as well as their fluid intake, which reduced their enjoyment of daily life. “I used to love eating all kinds of food, and food was like a salvation to me, but now, ‘I can’t eat what I want, and I can’t drink what I want,’ it’s so painful.” (P1) Finally, participants’ range of activities was restricted. The fixed dialysis schedule limited mobility, contributing to a loss of autonomy and control and increasing feelings of inferiority, anxiety, and fear. “In the past few months, I’ve gained over 30 pounds and can barely move. I have to use a wheelchair. Diabetes-related kidney disease requires me to restrict my diet, so how can I go out to eat? How can I go anywhere when I can’t move?” (P5) These lifestyle disruptions challenged participants’ autonomy and increased their dependence on others in daily life.

*Role transformation*. MHD treatment was perceived to alter or disrupt previously established social roles. 10 participants mentioned that they had shifted from being “contributors” in their families to “dependents,” which caused strong feelings of guilt and psychological burden. “I used to take care of everything at home, but now I have to rely on others for everything, and it doesn’t feel right.” (P1) “I feel like I’m a burden to my family. I can’t express it, but it’s really hard to bear.” (P10) In terms of work roles, limitations on or exit from the workforce also damaged participants’ self-esteem and raised doubts about their own value. “Since I stopped working, I feel like I’m useless.” (P7) Role changes affected participants’ perceptions of their own value and were associated with emotional distress.

*Reduced social participation*. Participants described reduced social participation as a consequence of physical limitations, dialysis schedules, and changes in daily routines. Unlike social withdrawal associated with worsening demoralization, reduced social participation primarily reflected fewer opportunities for social engagement due to illness-related restrictions. Among these accounts, reduced participation was primarily associated with reduced physical capacity rather than an intentional decision to distance themselves from others. “My health isn’t good, and I don’t feel like going out. Slowly, I’ve lost contact with friends.” (P8) “Now I rarely go out. I sit in a wheelchair, and my family pushes me out for fresh air. I feel like I’m a waste, unable to walk steadily.” (P11) Eight participants also described concerns about being viewed differently by others or becoming a burden, which further affected their willingness to maintain previous social interactions. These experiences reflected the influence of illness-related limitations and emotional concerns on participants’ social engagement during long-term dialysis.

## Impacts of demoralization syndrome

Participants described a range of psychological and behavioral changes associated with demoralization, including persistent negative emotions, social withdrawal, and reduced quality of life with difficulties in maintaining treatment-related behaviors. These experiences were described in the context of prolonged exposure to dialysis-related stressors and declining perceived ability to cope with ongoing challenges.

*Accumulation of negative emotions*. 10 participants described experiencing persistent low mood, anxiety, irritability, or emotional numbness under the prolonged pressures of long-term dialysis. Six participants emphasized that these emotions were not transient fluctuations but rather a persistent psychological state that was difficult to relieve. “It’s not that I felt bad for just a day or two, it’s like something has been weighing on me, and I just can’t seem to feel relaxed.” (P4) “Sometimes, nothing happened, but I still feel restless and exhausted.” (P11) Three participants described becoming less interested or less responsive to daily activities. Four participants also described negative evaluations of themselves and their situations, reflecting feelings of worthlessness and hopelessness associated with prolonged demoralization. “Sometimes, I wonder if my existence is just a burden to others, and the meaning of my life is only to cause trouble to others! Maybe I shouldn’t even be alive.” (P10) These experiences suggested that prolonged demoralization was accompanied by sustained emotional distress and reduced perceived ability to cope with ongoing challenges. Some of these experiences may overlap with depressive symptoms. However, participants’ narratives also emphasized helplessness, loss of meaning, reduced perceived control, and difficulties coping with ongoing illness-related stressors, which are central features of demoralization.

*Social withdrawal*. In contrast to reduced social participation caused by treatment burden, social withdrawal represented a psychological response that emerged in the context of prolonged demoralization. Participants described actively distancing themselves from others due to perceived stigma, fear of being a burden, reduced self-worth, or a belief that social interaction had lost meaning. 11 participants reported a significant decrease in their willingness to engage in social activities due to low mood and diminished self-worth, which gradually led to reduced contact with family, friends, and the broader community. “When my friends call, I sometimes make excuses not to go, because it just doesn’t seem meaningful.” (P8) “I used to often have meals with colleagues or go shopping, but now I have dialysis three times a week. After dialysis, I’m so tired I don’t want to move. I’m also afraid of others asking about the fistula on my arm, so I gradually stopped contacting them and became lazy to even answer the phone.” (P10) At the same time, five participants became more sensitive to their disease identity, fearing pity or being treated differently by others, and chose to actively withdraw from social situations. “When people ask about my health, I get really annoyed, so I just avoid seeing people.” (P9) “After being ill for so long, my family and friends have become numb. No one focuses their attention on a chronic patient anymore.” (P11) These accounts indicated reduced willingness to maintain interpersonal interactions and increased feelings of loneliness.

*Decline in quality of life and treatment adherence*. Participants described substantial effects of demoralization on their perceived quality of life, as well as difficulties in maintaining treatment-related behaviors. Four participants mentioned that, due to prolonged emotional distress, they could no longer pursue their ideals and values as they had previously and described how their lives had gradually lost a sense of purpose and meaning. They reported reduced interest and engagement in daily activities. “I just live day by day, not really having anything to look forward to.” (P8) “It feels like dialysis is the only thing left in my life; I can’t get excited about anything else.” (P14) Moreover, seven participants described difficulties maintaining treatment-related behaviors during periods of low mood and reduced hope. Five participants stated that they felt less motivated to follow dietary restrictions, manage their lifestyle, or monitor their health during periods of low mood or diminished hope for the future. “Sometimes I think, what’s the point of being so strict? What’s the use?” (P5) “The doctor asked me to do fist clenching exercises to protect the fistula every day, but sometimes I forget when I get home, it just feels like a hassle.” (P6) Participants’ accounts suggested that emotional distress was associated with reduced motivation and engagement in some disease-management behaviors.

## Coping strategies

Participants adopted different coping strategies in response to ongoing stressors. These strategies varied across individuals and over time. Rather than representing a single adaptive or maladaptive pattern, these coping responses reflected differences in participants’ perceived control, emotional resources, and ability to respond to long-term dialysis-related challenges. This theme includes three subthemes: helpless resignation, avoidance and concealment, and active coping and behavioral activation.

*Helpless resignation*. An important finding was that apparent acceptance of dialysis did not necessarily represent adaptive psychological acceptance. Six participants, after long-term treatment and repeated exposure to dialysis-related stressors, gradually developed a pattern of helpless resignation. Although these participants appeared to “accept” their condition, their narratives indicated that this was not an active psychological acceptance of illness, but rather a passive adjustment characterized by limited perceived choices and reduced expectations for the future. They did not truly embrace their disease, but instead passively compromised with the reality after prolonged exposure to illness-related challenges. This coping pattern was often accompanied by feelings of powerlessness, emotional suppression, and a reduced sense of control. “It’s not that I’ve come to terms with it; it’s that there’s no choice. What else can I do? If I don’t do dialysis, I won’t survive.” (P6) “I slowly stopped struggling. It’s all the same now, no point in thinking about it.” (P11) In this coping strategy, participants intentionally lower their expectations for the future to avoid further disappointment and frustration. They focus more on maintaining the status quo rather than improving their lives. “I can’t think about the future now, I just take it day by day. Thinking too much makes me feel worse.” (P2) These accounts reflected resignation following prolonged illness-related stress and repeated difficulties in adaptation, rather than adaptive acceptance of the disease.

*Avoidance and concealment*. Five participants coped with the psychological pressure of their disease by concealing it. They tended to avoid talking about their condition or intentionally downplay their illness identity in family and social interactions to reduce discomfort from being observed, pitied, or treated differently. “I don’t really want to tell others that I’m on dialysis. It’s not helpful, and it feels weird when people look at me.” (P9) “Sometimes, when my family asks, I don’t want to talk much. The more I talk, the more upset I feel.” (P4) In addition, three participants temporarily alleviated psychological burden by avoiding disease-related information or situations, but this also reduced opportunities for emotional expression and support. “If I don’t think about it or ask, life seems to be a little easier, otherwise the more I know, the more scared I get.” (P13) This coping strategy provided short-term emotional relief but could also limit opportunities for seeking information, expressing emotions, and obtaining social support.

*Active coping and behavioral activation*. Eight participants gradually adopted more active coping strategies in the process of adapting to their illness. After acknowledging the long-term nature of dialysis, these participants attempted to adjust their perceptions, lifestyles, and daily behaviors to maintain involvement in life activities. One participant described the process of accepting the chronic nature of dialysis and adjusting their mindset: “Dialysis cannot be changed; I can only adjust my mindset.” (P3) Another participant similarly expressed that accepting the illness helped them re-engage with daily life: “After I figured it out, the disease is the disease, but life still has to go on. If I don’t do anything, I just end up overthinking.” (P7) Beyond cognitive adjustment, these participants also made deliberate efforts to maintain daily activities and develop interests within the limits of their physical condition, reducing excessive focus on the disease. For example, one participant described continuing household activities despite physical limitations: “As long as my physical condition allows, I still do some housework.” (P1) Another participant highlighted the importance of staying engaged in daily life outside dialysis: “During the time outside of dialysis, I try to keep myself busy and not let my life be reduced to just seeing doctors.” (P15) These accounts illustrate several forms of active coping, including cognitive adjustment, maintenance of daily activities, and deliberate efforts to preserve engagement and a sense of normality. Although such accounts were reported by eight participants, the qualitative evidence did not permit systematic comparison of active coping across participants.

## Stress-buffering resources

During long-term hemodialysis, participants described various support resources that helped them manage illness-related challenges. These resources included informational support, emotional support, and policy-related support. However, participants reported differences in the availability, accessibility, and adequacy of these resources.

*Informational support*. Nine participants expressed a strong need for clear and continuous informational support during the interviews. Despite undergoing long-term dialysis treatment, four participants still lacked a comprehensive understanding of disease progression, treatment expectations, and key aspects of self-management, leading to persistent anxiety and confusion. “I’ve been doing dialysis for so long, but there are still many things I don’t fully understand. I just know to come for dialysis on time, but I’m unsure about the future.” (P8) “Sometimes when I feel unwell, I’m not sure if it’s normal, and I don’t know who to ask.” (P3) Three participants indicated that existing information mainly focuses on procedural aspects, while explanations regarding the long-term impact on daily life, psychological changes, and future planning are relatively insufficient. “The doctor talks more about indicators and data, but what we really want to know is how long we will live like this and whether it will keep getting worse.” (P14) These accounts suggested that insufficient or unclear information limited participants’ understanding of their illness trajectory and affected their ability to develop realistic expectations and coping plans.

*Emotional support*. Regarding emotional support, participants expressed a desire to be understood and listened to. Eight participants reported that although they had family members by their side, they still found it difficult to express their true feelings because they feared burdening others or causing unnecessary worry. “There are some things I don’t dare to tell my family because it makes them feel bad, and I feel guilty for making them worry.” (P6) “A lot of times, I feel very depressed, but I don’t know who I can talk to.” (P1) At the same time, three participants (P2, P5, and P10) felt that healthcare professionals focused more on the treatment itself and were relatively less attentive to their emotional changes and psychological state, leaving their emotional needs unrecognized. “There are people who manage my physical issues, but no one seems to ask about my mental state.” (P10) These experiences reflected unmet emotional needs and limited opportunities for psychological expression and emotional communication during long-term dialysis care.

*Policy support*. In terms of institutional and policy support, participants expressed a need for economic assistance and social security. Long-term dialysis not only imposed a physical burden but also continuously affected family finances and participants’ ability to participate in social activities. “Dialysis itself is expensive, and there are many hidden costs as well. After a long time, the pressure is really overwhelming.” (P12) “Some subsidies are available, but the application process is complex, and I’m not sure whether I meet the eligibility criteria.” (P5) Four participants (P5, P6, P9, and P12) specifically mentioned that dialysis scheduling, work support policies, and access to social welfare directly influenced their quality of life and engagement in treatment-related behaviors. “If there were more understanding and support at work, maybe I wouldn’t feel so mentally exhausted.” (P9) These descriptions highlighted that institutional and policy-related support represented an important external resource, but difficulties in accessibility and implementation limited the potential benefits of such support.

## Discussion

This study used a deductive, framework-informed thematic analysis guided by the SPM to explore experiences of DS among patients receiving MHD. The findings should not be interpreted as suggesting that common dialysis-related stressors are themselves specific indicators of demoralization. Rather, the analytic contribution of this study lies in illustrating how persistent physical burden, uncertainty, role disruption, and social restriction may become demoralizing when they erode patients’ sense of meaning, perceived ability to cope, agency, and expectations for a meaningful future. Two distinctions were particularly informative within this framework. First, reduced social participation reflected externally imposed restrictions related to illness and dialysis, whereas social withdrawal represented a more intentional behavioral response associated with perceived burden, diminished self-worth, and loss of meaning. Second, apparent acceptance of dialysis did not necessarily indicate adaptive acceptance; for some participants, it reflected helpless resignation and passive compromise under conditions of limited perceived alternatives. Together, these findings suggest that the psychological specificity of DS lies less in the presence of dialysis-related stressors than in how these stressors are interpreted and incorporated into patients’ perceptions of self, coping capacity, meaning, and future possibility.

The findings also provide a link between the SPM and multidimensional perspectives on resilience, functioning, and adaptation. From this perspective, adaptation to long-term dialysis involves more than reducing psychological distress; it also entails preserving meaningful functioning, agency, social engagement, and a sense of future possibility in the face of persistent treatment demands. The present findings suggest that perceived control may be one mechanism connecting stress exposure with adaptation: when participants retained a sense of influence over daily activities and valued roles, they were more likely to describe active coping and continued engagement, whereas prolonged stress and limited coping resources were associated with helpless resignation, social withdrawal, and reduced future orientation. These patterns suggest that resilience may be reflected not only in the capacity to withstand stress, but also in the ability to maintain or restore meaningful roles, agency, and engagement despite ongoing adversity. Thus, the SPM helps explain the accumulation and contextual effects of stress, while resilience and perceived-control perspectives highlight how these processes may shape patients’ functioning and adaptive responses over time.

### The role of multidimensional stressors in shaping DS experiences among patients receiving MHD

Although dialysis-related physical discomfort, disease uncertainty, lifestyle disruption, role transformation, and reduced social participation are widely reported among patients receiving maintenance hemodialysis, their relevance to DS in the present study lies in how patients interpreted these experiences and integrated them into their perceptions of self, coping capacity, and future possibilities (Lin et al., 2025).

According to the SPM (Pearlin et al., 1990), primary stressors refer to direct challenges arising from adverse life circumstances, whereas secondary stressors emerge when these initial challenges interfere with individuals’ social roles, personal goals, and available resources. In the context of MHD, dialysis-related physical symptoms and uncertainty regarding disease outcomes can be considered primary stressors. However, our findings indicate that the psychological consequences of these stressors extend beyond physical discomfort or treatment inconvenience. When patients are unable to maintain valued activities, previous social roles, or desired lifestyles because of dialysis-related limitations, they may gradually experience a reduced sense of self-worth, loss of meaning, and diminished perceived control, which may in turn weaken their perceived capacity to cope and their ability to envision a meaningful future (Elias et al., 2025).

For example, persistent physical symptoms, dietary restrictions, and treatment-related demands may limit patients’ participation in work, family responsibilities, and social activities (Al-Naamani et al., 2024). Although these burdens are well established in the dialysis literature, their significance in the present study lay in how they challenged valued roles, identity, and sense of purpose. Participants described feeling unable to contribute to their families or maintain their previous way of life. These limitations were therefore experienced not only as losses of functional ability but also as threats to identity and purpose, which may further reinforce feelings of helplessness and perceived inability to cope with ongoing and largely uncontrollable demands. Thus, the pathway from common dialysis-related burdens to DS may involve a shift from experiencing illness-related limitation to perceiving oneself as unable to cope, unable to maintain what gives life meaning, and increasingly unable to envision a meaningful future (Milders et al., 2025).

Furthermore, disease uncertainty and the long-term nature of dialysis may contribute not only to emotional distress but also to a foreshortened sense of the future and a diminished ability to envision meaningful future possibilities. Unlike general distress associated with illness burden, demoralization involves a more profound disruption in patients’ perceived ability to cope and maintain a meaningful future, often accompanied by helplessness and loss of purpose (Ye et al., 2025). Thus, uncertainty may become particularly relevant to DS when patients no longer perceive the future as something they can actively shape or look forward to, rather than simply feeling worried about disease progression. Thus, the contribution of dialysis-related stressors to DS lies not merely in the objective severity of these challenges but in how patients interpret and respond to them.

Furthermore, reduced social participation and social withdrawal may represent different processes within the development of DS. Reduced social participation was primarily associated with physical limitations and treatment-related restrictions, whereas social withdrawal reflected a more active behavioral response involving perceived burden, diminished self-worth, and reduced connection with others (Sluiter et al., 2024; Diao et al., 2025). This distinction further highlights that DS is not simply characterized by reduced activity or social difficulties, but by the meanings patients attach to these experiences and their perceived ability to maintain agency, cope with life changes, and preserve a meaningful future.

Clinically, these findings highlight the importance of assessing DS from a multidimensional perspective. Healthcare professionals should not only evaluate depressive symptoms or emotional distress but also consider changes in patients’ roles, perceived control, sense of meaning, and future expectations when identifying patients at risk of demoralization.

### Psychological consequences, coping resource depletion, and coping strategies in DS among patients receiving MHD

Participants described substantial psychological and behavioral consequences associated with DS, including helplessness, social withdrawal, diminished motivation, and difficulties maintaining a sense of purpose and agency. According to the SPM, continuous exposure to stressors may gradually consume available coping resources, thereby reducing individuals’ ability to effectively manage subsequent challenges (Pearlin et al., 1981). In this study, repeated experiences of physical discomfort, uncertainty, role disruption, and social limitations appeared to place sustained demands on patients’ psychological resources. When patients experienced difficulty restoring a sense of control or adapting to changing circumstances, they described feelings of helplessness, emotional exhaustion, and reduced motivation. These experiences may reflect patients’ perceptions that their available coping capacities were insufficient to address ongoing illness-related demands.

The impact of DS was particularly evident in patients’ changing perceptions of identity and personal value. Many participants described a transition from being independent individuals who contributed to their families and society to becoming individuals who depended on others for care. This perceived shift in identity may weaken self-esteem and challenge patients’ understanding of their own worth, thereby contributing to loss of meaning and a diminished sense of agency. Such experiences are consistent with previous research on chronic illness adaptation, which has demonstrated that illness-related role disruption and dependency can threaten patients’ sense of autonomy and personal meaning (Cuesta-Benjumea et al., 2023). The findings also revealed that coping responses among MHD patients were diverse and dynamic, including helpless resignation, avoidance and concealment, and active coping. Importantly, these coping patterns should not be interpreted simply as adaptive or maladaptive behaviors; rather, they may represent different manifestations of patients’ perceived coping capacity and sense of control. Patients who adopted helpless resignation often described accepting dialysis because they perceived limited alternatives rather than because they had achieved genuine psychological adjustment (Xie et al., 2023). In this context, resignation may indicate not acceptance of the illness itself, but a perceived inability to cope with circumstances experienced as largely uncontrollable. Similarly, avoidance and concealment temporarily reduced emotional discomfort but also restricted opportunities for emotional expression and support seeking (Cai et al., 2025; Huang et al., 2025). These responses may therefore be particularly relevant to demoralization when participants experience limited agency and perceive few alternatives, rather than simply reflecting ineffective coping.

From the perspective of the SPM, these coping patterns may reflect the consequences of prolonged stress exposure and resource depletion. When patients repeatedly encounter uncontrollable stressors without sufficient internal or external resources, maintaining active problem-solving strategies may become increasingly difficult. In contrast, eight participants described active coping strategies, such as adjusting daily routines, maintaining meaningful activities, and reframing dialysis as part of life rather than the end of life, and these accounts appeared to reflect a greater sense of agency and purpose (Kapadi et al., 2025). This finding tentatively suggests that active coping may be associated with the preservation of identity, meaning, and perceived control; however, this interpretation should remain exploratory given the limited number of participants providing such accounts. This interpretation is consistent with previous studies indicating that meaning-oriented coping, cognitive adaptation, and preservation of autonomy may facilitate psychological adjustment in serious illness (Natale et al., 2019). Rather than merely reducing distress, adaptive coping may help patients reconstruct meaning within the context of lifelong dialysis and maintain a sense that their lives remain valuable and controllable.

Nevertheless, coping strategies are influenced by individual and sociocultural factors (Subramanian et al., 2017). In the Chinese family-centered cultural context, patients may prioritize maintaining family harmony and avoiding placing an additional burden on family members. Consequently, some patients may conceal illness-related emotions or avoid discussing their distress with family members, as reflected in participants’ accounts. Although such avoidance and concealment may temporarily reduce interpersonal tension, they may also limit opportunities for emotional expression and support, contributing to patients’ perceived lack of emotional support (Xiong and An, 2025). Thus, these coping responses may reflect not only individual avoidance but also culturally shaped efforts to protect family relationships. When such strategies simultaneously limit emotional disclosure and support seeking, they may further weaken perceived coping resources and reinforce the isolation, helplessness, and loss of meaning associated with demoralization. Clinically, routine assessment of DS using validated instruments such as DS-II may help identify patients experiencing loss of meaning, helplessness, and reduced future expectations (Huang et al., 2025). Interventions incorporating behavioral activation, cognitive restructuring, and supportive counseling may represent potential approaches based on previous evidence (Ciharova et al., 2021); however, their effectiveness specifically for MHD patients with DS requires further empirical evaluation.

### The buffering role and limitations of social support in shaping DS experiences among MHD patients

Consistent with the SPM, this study identified informational, emotional, and policy-related support as important resources that may buffer the psychological consequences of chronic illness stress. However, participants also described substantial unmet needs, suggesting that the availability of support alone may not always translate into effective stress buffering. The SPM emphasizes that social resources can reduce the negative effects of stress when they are accessible, adequate, and responsive to individuals’ specific needs. In this study, the buffering function of support appeared to depend not only on whether resources existed but also on whether patients perceived them as meaningful and helpful, particularly in restoring a sense of connection, coping capacity, control, and future possibility. For example, although many participants received support from family members, some hesitated to disclose emotional distress because they feared becoming a burden. As a result, available family support did not always fully address their psychological needs.

Similarly, some participants perceived that healthcare interactions focused primarily on physiological indicators, such as laboratory values and dialysis-related management, whereas their emotional experiences received less attention. This mismatch between biomedical care priorities and patients’ psychological needs may limit the potential buffering effect of healthcare support (Nataatmadja et al., 2021). These findings highlight that effective support requires not only the provision of information or assistance but also recognition of patients’ emotional experiences and individual concerns (de Jong et al., 2021). Informational support was particularly important because uncertainty regarding disease progression and future life expectations was a prominent stressor. When patients lacked sufficient understanding of their condition, treatment expectations, and self-management strategies, uncertainty remained unresolved and may have continued to influence feelings of helplessness (Xie et al., 2023). Therefore, information should extend beyond technical aspects of dialysis management and include guidance regarding psychological adaptation, lifestyle adjustment, and future planning to strengthen patients’ perceived control and ability to maintain a meaningful future orientation.

Emotional and social support may also play a critical role in protecting patients’ sense of belonging and personal value. When emotional needs remain unrecognized, patients may become increasingly isolated and may interpret illness-related limitations as evidence of personal loss or reduced worth. Conversely, supportive interactions that validate patients’ experiences may help restore connection, meaning, perceived control, and adaptive functioning (Sluiter et al., 2024).

Policy-related resources represented another important but often insufficiently addressed domain. Financial burden, limited flexibility in dialysis arrangements, and difficulties accessing supportive policies were described as additional sources of stress. Although these structural stressors are not specific to DS, they may become relevant to demoralization when they further restrict patients’ perceived options, autonomy, and ability to envision a manageable future. Although these structural factors extend beyond the direct scope of individual psychological interventions, they represent important contextual conditions that shape patients’ ability to adapt to long-term dialysis (Novick et al., 2024).

Based on these findings, clinical practice should emphasize the development of multidimensional support systems. Interventions directly informed by participants’ experiences include improving individualized health education, strengthening nurse–patient communication, and creating opportunities for emotional expression. Furthermore, integrating emotional support and meaning-oriented interventions into routine dialysis care may help patients maintain a sense of identity, connection, and purpose during long-term treatment. Importantly, such approaches should target not only general psychological distress, but also the core features of demoralization, including loss of meaning, perceived inability to cope, and diminished expectations for a meaningful future. Given the potential overlap with depression and the presence of passive suicidal ideation in some participants, psychological assessment should also incorporate appropriate monitoring of depressive symptoms and suicide risk, with established referral pathways when clinically indicated.

## Limitations

This study has several limitations. First, participation-related self-selection may have influenced the composition of the qualitative sample. Of the 36 invited patients, 20 declined participation, including eight because of physical discomfort and seven because they were unwilling to discuss personal experiences. Although maximum-variation sampling was used to seek heterogeneity across key characteristics, no formal statistical comparison between participants and decliners was conducted. Therefore, the final sample may have underrepresented patients who were less physically able to participate or less willing to discuss distressing experiences. This self-selection may limit the transferability of the findings to the broader population of patients with moderate-to-severe demoralization.

Second, each participant completed only one interview, which may have limited exploration of changes in DS experiences over time. The use of the SPM as the primary framework may also have influenced the organization and interpretation of the findings, despite reflexive discussions throughout the analysis.

Third, the single-center design and reliance on patient perspectives alone may limit the transferability and comprehensiveness of the findings. Depression was not independently assessed in the parent survey or during the qualitative study; therefore, depressive symptoms or comorbid depression could not be excluded. Some reported experiences, particularly low mood, reduced interest, hopelessness, social withdrawal, and suicidal ideation, may overlap with depression, and the present study therefore cannot determine whether these experiences are specific to demoralization. Although the participants represented a relatively wide range of DS-II scores (10–27), including 10 participants with moderate demoralization (10–19) and 5 with severe demoralization (≥20), the study was not designed to compare experiences across severity levels. Therefore, potential differences in experiences between moderate and severe demoralization remain unexamined. Finally, because the interviews were conducted in Chinese and quotations were translated into English, some contextual meanings may have been lost, and the cross-sectional nature of the study limits the ability to establish temporal or causal relationships. Future multicenter longitudinal and mixed-method studies should examine demoralization and depression concurrently and further explore how perceived control, resilience, and supportive resources influence adaptation and functioning over time.

## Conclusion

This study explored the experiences of DS among patients receiving MHD based on the SPM. The findings suggest that DS is a complex psychological experience shaped by the interaction of dialysis-related stressors, coping responses, and stress-buffering resources. These findings provide insights into patients’ psychological needs and may inform future interventions, while further multicenter longitudinal and mixed-method research integrating the perspectives of patients, caregivers, and healthcare professionals is needed to deepen understanding of DS.

## Data Availability

The datasets generated and analyzed during the current study are not publicly available due to ethical restrictions and concerns regarding participant confidentiality. The qualitative interview transcripts contain sensitive personal health information and cannot be shared publicly. Requests to access the datasets should be directed to Jianfang Bao, 3178817525@qq.com.
