# Supplementary material for: Experiences of moderate-to-severe demoralization syndrome among patients receiving maintenance hemodialysis: a qualitative study
Source: Front Psychol. 2026 Oct 15;17:1931026. doi: 10.3389/fpsyg.2026.1931026 (PMC13716865; doi:10.3389/fpsyg.2026.1931026)
Supplement: Supplementary file 1 [file Data_Sheet_1.pdf]

**Supplementary File S1. Semi-Structured Interview Guide**

**The interviewer stated:**
"Thank you for participating in this study. We would like to understand your experiences and feelings related to living with dialysis. There are no right or wrong answers. We hope that you can freely share your thoughts, feelings, and experiences. All information you provide will be kept confidential and used only for research purposes."

**1、Could you share your experiences since starting dialysis? How has dialysis changed your life?**

**Probing questions:**
How did you feel when you first started dialysis?
What changes have occurred in your daily life, family roles, work, or social activities?
How do you perceive these changes?

**2、During your dialysis treatments, what have been the most helpless, frustrating, or demoralizing moments? Could you describe these feelings in more detail?**

**Probing questions:**
Can you recall a specific situation that made you feel this way?
What thoughts or emotions occurred at that time?
How did these experiences influence your view of yourself or your future?

**3、How have these feelings impacted your life? How do you cope with and manage these psychological reactions?**

**Probing questions:**
Have these feelings influenced your daily activities or relationships?
What strategies have you used to deal with these emotional experiences?
Were these strategies helpful?

**4、What do you believe these feelings are related to?**

**Probing questions:**
Are these feelings related to physical symptoms, dialysis treatment, disease uncertainty, or lifestyle changes?
Are there any experiences or events that particularly contributed to these feelings?

**5、At this stage, what challenges are you facing? What is the biggest challenge for you?**

**Probing questions:**
What aspects of dialysis or illness are most difficult for you to accept?
How do these challenges affect your future plans or expectations?

**6、How do you think the people around you (family, friends, or healthcare professionals) can help? Are there any other thoughts or feelings you would like to share?**

**Probing questions:**
What type of support do you need most?
How do you perceive the support you currently receive?
Is there anything you wish others could better understand?

**7、What advice would you offer to those who are just starting dialysis? What**

**would you like to say to them?**
**Probing questions:**
Based on your experience, what should new patients prepare for?
What factors may help them adapt better to dialysis?
**8、Is there anything else you would like to add or share that we have not covered?**
**Probing questions:**
Are there any experiences related to dialysis or psychological changes that you think are important?
Is there anything you would like healthcare professionals to know?
**The interviewer stated:**
“Thank you very much for sharing your experiences and thoughts. Your information is valuable for understanding the psychological experiences of patients undergoing maintenance hemodialysis and improving supportive care for future patients.”
